# Grafting photochromic spiropyran polymer brushes on graphene oxide surfaces *via* surface-initiated ring-opening metathesis polymerization

**DOI:** 10.1039/d3ra08076e

**Published:** 2024-01-25

**Authors:** BangSen Li, Wenya Zhu, Jinrui Liu, Shishu Sun, Yan Zhang, DaShuai Zhang, Chen Li, Jianjun Shi, Zaifeng Shi

**Affiliations:** a Collage of Chemistry and Chemical Engineering, Hainan Normal University Haikou 571158 China zaifengshi@163.com stone0801@163.com; b Key Laboratory of Water Pollution Treatment and Resource Reuse of Hainan Province Haikou 571158 China; c Key Laboratory of Water Pollution Treatment and Control of Haikou City Haikou 571158 China

## Abstract

A practical “grafting-from” strategy is described to grow photochromic polymer brushes bearing spiropyran (SP) functional groups on graphene oxide (GO) surfaces *via* surface-initiated ring-opening metathesis polymerization (SI-ROMP). The Grubbs II catalyst was fixed on the GO surface, and the norbornene derivatives functionalized using spiropyran were synthesized from this active site *via* the ROMP method. The results indicated that the spiropyran-modified polymer brushes were obtained on the GO surface in the form of thin films. The solubility of GO modified by spiropyran polymers (GO-SPs) in organic solvents was significantly improved. The GO-SPs exhibited excellent photochromic properties, including fast coloration/decoloration. The modified GO with an isomeric structure was colored in 90 s under ultraviolet irradiation and decolored in 360 s under white light. The fading kinetic rate in the dark was slow and the kinetic attenuation curve followed bi-exponential decay. The GO-SP composite materials took more than 2 h to return to thermodynamically stable forms. The reversible change in the water contact angle reached 8° after continuous cycling with ultraviolet and visible light. GO-SP maintained its photochromic performance and possessed excellent fatigue resistance after more than six successive UV/light cycles. This work describes a practical strategy for the preparation of photochromic polymer brush modified GO composite materials and extends the applications of GO in photochromic materials.

## Abstract 2

A practical “grafting-from” strategy is described to grow photochromic polymer brushes bearing spiropyran (SP) functional groups on graphene oxide (GO) surfaces *via* surface-initiated ring-opening metathesis polymerization (SI-ROMP).

## Graphical abstract

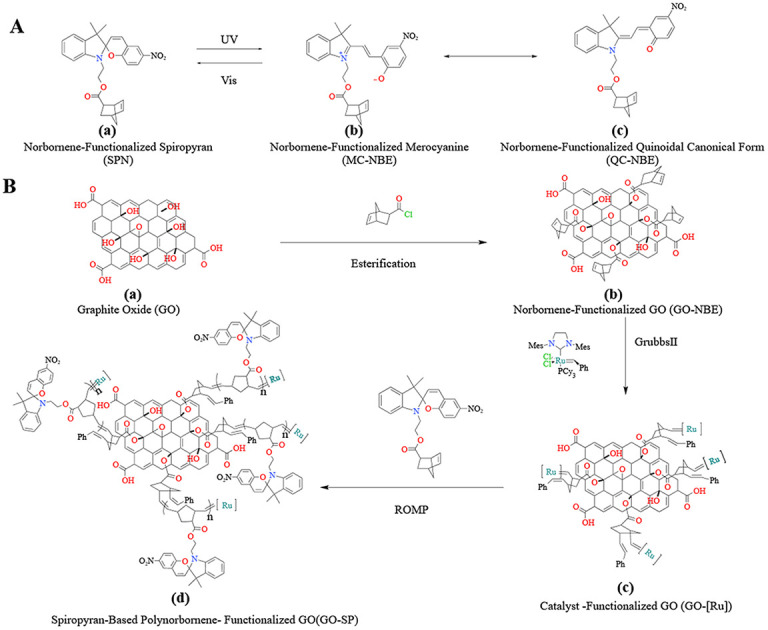

## 1. Introduction

The surface of graphene oxide (GO) contains substantial hydroxyl, carboxyl, and epoxide groups. These functional groups provide great possibilities for covalent and non-covalent approaches to the modification of GO materials.^1,2^ The surface modification of GO has been widely applied in the fields of bioimaging,^3–7^ drug delivery,^8–10^ material self-healing,^11,12^ and catalysis.^13^ The “grafting-from” approach is an attractive covalent modification strategy based on surface-initiated grafted polymers. This method requires anchoring of the initiated sites on the surface of substrates and achieving the growth of polymer chains under the action of the corresponding catalysts. The advantages of the “grafting-from” method include less steric hindrance and restriction on the growth of polymer chains.^14^ Hitherto, controlled surface-initiated polymerization techniques, namely atom transfer radical polymerization,^15–17^ reversible addition–fragmentation chain-transfer polymerization,^18–20^ ring-opening metathesis polymerization,^21,22^ and ring-opening polymerization^23^ were applied in the research of grafting functionalized polymers on GO surfaces.

**Fig. 1:**
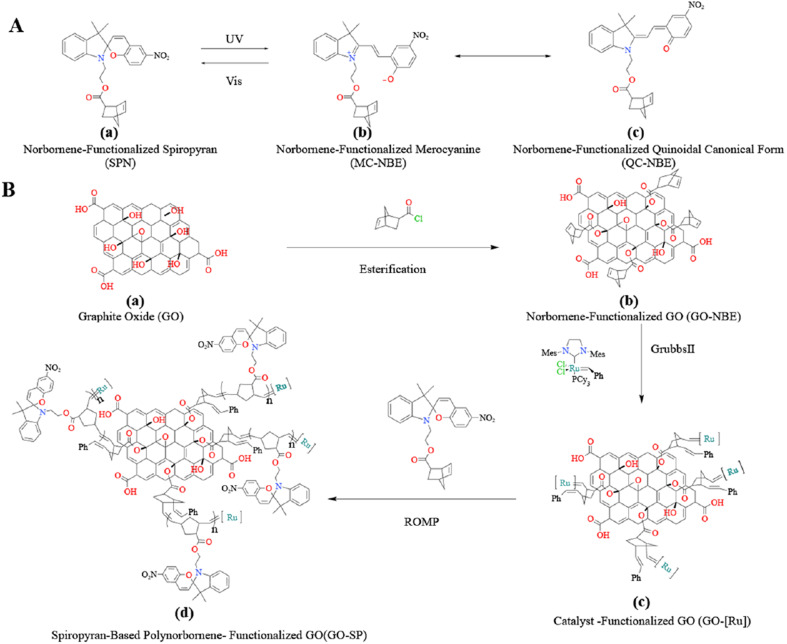
(A) The isomeric molecular structure of spiropyran under UV irradiation and (B) process for the synthesis of GO-SP by the SI-ROMP method.

The norbornene (NBE) polymers and their derivatives initiated by Grubbs catalyst *via* living ROMP have good stability, and the functional groups of norbornenes provide enormous potential and possibilities for the design of various functional polymers. Functionalized polynorbornenes and their derivatives have been applied to materials for property improvement by SI-ROMP.^24,25^ Recently, our group reported that fluorinated norbornenes and polyethylene glycol-substituted norbornenes as monomers were polymerized on GO surfaces.^22^ In our study, the modified GO showed good dispersion in dichloromethane due to the hydrophobicity of the grafted polymer and copolymer, and the modified GO had better adsorption resistance to large molecular weight proteins. Photochromic spiropyran materials have been widely used in the fields of information storage components,^26^ intelligent decorative materials,^27^ and anti-counterfeiting.^28^ The C–O bond of the spiropyran (SP) form undergoes cleavage and the SP form transforms into a ring-opened merocyanine (MC) form under the condition of ultraviolet irradiation. When the MC form is irradiated by visible light, it will undergo a reversible ring-closed process and transform into the SP form (as shown in Fig. 1a). This reversible change in the chemical structure provides a foundation for the design of photochromic smart materials, and the synthesis of SP derivatives has attracted extensive attention for applications involving material modifications, such as photic driving,^29–31^ sensing,^32^ wettability^33,34^ and so on.

In this work, the norbornene-modified GO (GO-NBE) was prepared by esterification on its –OH site. The active macromolecular initiator (GO-[Ru]) was generated under the condition of the ROMP reaction between GO-NBE and Grubbs II. The growth of SP-functionalized norbornene (SPN) was achieved on the surface of GO layers. The modified GO exhibits better hydrophobicity and uniform dispersion in CH_2_Cl_2_. The relationship between photochromic properties and the structure of the polymers was analyzed. Novel photochromic GO-SP nanomaterials were prepared with high photostability and fast coloring/decoloring cycles. This synthesis strategy of monomer design and polymer grafting broadens the scope of GO applications, and the obtained materials have application potential in intelligent response nanomaterials.

## 2. Experimental section

### 2.1. Materials

Graphene oxide (GO), high-performance liquid chromatography-grade tetrahydrofuran (HPLC-THF), 5-norbornene-2-carboxylic acid (NBA), and triethylamine were purchased from Macklin. Pyridine and (1,3-bis-(2,4,6-trimethylphenyl)-2-imidazolidinylidene) dichloro (phenyl-methylene)(tricyclohexylphosphine) ruthenium (Grubbs II) were purchased from Aladdin. GO was thoroughly cleaned and dried before testing to eliminate the interference of nitric acid residue. All other reagents and solvents were purchased from commercial suppliers and used as received unless otherwise noted. 1′-(2-Hydroxyethyl)3′,3′-dimethyl-6-nitrospiro (2*H*-1-benzopyran-2,2′-indole) (SP alcohol) was synthesized according to the literature procedure.^34^

### 2.2 Synthesis of SP-functionalized norbornene (SPN) monomers

The mixture of NBA (1.38 g, 10 mmol) and thionyl chloride (15 mL) was heated at 70 °C for 1 h under a nitrogen atmosphere. After vacuum distillation of excess thionyl chloride, the resulting liquid was used immediately without further purification. SP alcohol (5 mmol) and pyridine (0.475 g, 6 mmol) were dissolved in freshly distilled THF (25 mL) in a nitrogen atmosphere. 5-Norbornyl chloride (0.783 g, 5 mmol) was dissolved in anhydrous THF (10 mL) and added to the reaction solution. The reaction mixture was stirred for 24 hours at room temperature. After filtering the solid residue and concentrating the reaction solution, the gray solid was separated by silica gel column chromatography using hexane/ethyl acetate (10 : 1 v/v) as the eluent. Yield: 1.2 g (50%). ^1^H NMR (400 MHz, chloroform-*d*) *δ* (ppm): 8.02 (dq, *J* = 11.0, 2.3, 1.6 Hz, 4H), 7.21 (ddt, *J* = 7.7, 6.3, 1.2 Hz, 2H), 7.09 (dd, *J* = 7.3, 1.2 Hz, 2H), 6.95–6.85 (m, 4H), 6.79–6.67 (m, 4H), 6.19–6.02 (m, 3H), 5.98–5.83 (m, 3H), 4.33–4.06 (m, 4H), 3.58–3.32 (m, 4H), 3.14 (ddq, *J* = 10.6, 3.6, 1.7 Hz, 1H), 2.94–2.82 (m, 4H), 2.20–2.10 (m, 1H), 1.94–1.79 (m, 2H), 1.58 (s, 2H), 1.41 (dddd, *J* = 11.9, 10.4, 6.8, 1.8 Hz, 2H), 1.39–1.14 (m, 17H).

### 2.3 Synthetic procedure of norbornene modified GO (GO-NBE)

GO (85 mg) and THF (15 mL) were placed in a 50 mL round bottom flask and treated under ultrasonic dispersion for 1 h. Then, triethylamine (870 mL, 6 mmol) and 5-norbornene-2-acyl chloride (NBA, 0.783 g, 5 mmol) were added to the GO dispersion in an ice-water bath. Subsequently, the reaction mixtures were slowly heated to room temperature. After stirring for 24 h, the mixtures were centrifugated and washed 3 times with distilled water and THF. Then, the solid particles were dried in a vacuum at 30 °C for 12 h, and norbornene-modified GO was obtained.

### 2.4 Surface-initiated polymerization of GO-NBE

GO-NBE (20 mg) and THF (5 mL) were placed in a round bottom flask with the treatment of ultrasonic wave for 25 min. Then, Grubbs II (4 mg, 4.7 × 10^−3^ mmol) in 2 mL anhydrous THF was added to the reaction mixtures and the reaction was carried out under a nitrogen atmosphere for 30 min. Subsequently, the reaction mixture was separated by repeated centrifugation and resuspension with THF. The obtained GO-[Ru] and THF (5 mL) were placed in a round bottom flask under ultrasonic dispersion for 10 min. SPN (0.5 M) in 2 mL THF solution was added to the reaction flask and bubbled with nitrogen. After stirring for 30 min, the reaction mixture was centrifugated and washed 3 times with THF. The supernatant was added to methanol for the reprecipitation of modified GO. GO-SP nanoparticles were obtained after drying for 24 h in a vacuum at 30 °C.

### 2.5. Characterization

The nuclear magnetic resonance (^1^H-NMR) spectra were obtained using a Bruker AV 400 MHz NMR spectrometer with chloroform-*d* as a solvent. FTIR spectra were recorded on a Thermo Scientific Nicolet 6700 spectrometer at a resolution of 4 cm^−1^ under the wave number range of 4000–400 cm^−1^ and the average scans of over 32 times at room temperature. The samples were prepared using powder-pressed KBr pellets. XRD analysis was performed on an Ultima IV X-ray diffractometer (Rigaku, Japan) under the condition of 2*θ* range from 5 to 60°, 5°/min scan rate, and monochromatic Cu Ka source (*λ* = 0.154056 nm). Raman spectra were obtained using a HORIBA Scientific Lab RAM HR Evolution spectrometer with a 514 nm nano-argon ion laser. XPS spectra were obtained on a Thermo Scientific K-Alpha electron spectrometer with an Al-Kα source. Scanning electron microscopy (SEM) images were captured on a JSM-7100F instrument (JEOL, Japan) under an acceleration voltage of 5 kV. UV-vis absorption was performed using a UV-2007 spectrometer (Shimadzu) and UV-2006 spectrometer (Shimadzu). The ultraviolet light source was an ultraviolet flashlight (Tank556, 365 nm). The visible light source was a 40 W tungsten lamp passing through a 570 nm filter.

## 3. Results and discussion

### 3.1 Synthesis and structural characterizations of functionalized GO-SP by SI-ROMP

Grubbs II with high tolerance and selectivity of functional groups was considered to apply to the ROMP reaction. SP-modified norbornene derivatives as monomers were polymerized on the GO surface because of the high ring strain from the norbornene structure. The preparation process of GO-SP is shown in Fig. 1. First, GO-NBE was synthesized by the esterification between the –OH groups on the GO (Fig. 1B(a)) surface and 5-norbornene-2-acyl chloride (Fig. 1B(b)). Then, Grubbs II was fixed on GO-NBE using the ROMP reaction as the macromolecular initiator (GO-[Ru]) (Fig. 1B(c)). Here, the metal ruthenium carbenes were used as active sites during polymerization. Subsequently, the SPN monomers took out ROMP at the active GO-[Ru] sites to prepare the spiropyran-modified polynorbornene functionalized GO (GO-SP) (Fig. 1B(d)). There were four major procedures in the preparation of photochromic spiropyran polymer brushes on the GO surface: the synthesis of SP-modified norbornene derivatives; the esterification reaction between GO and excess 5-norbornene-2-acyl chloride for the preparation of norbornenyl initiator ligands on the GO surface; the synthesis of effective GO-[Ru] for the ROMP; the polymerization of SP-NBE monomers and the growth of polymer brushes on the GO surface. The SI-ROMP provided a practical solution to the functionalization of GO materials.

**Fig. 2:**
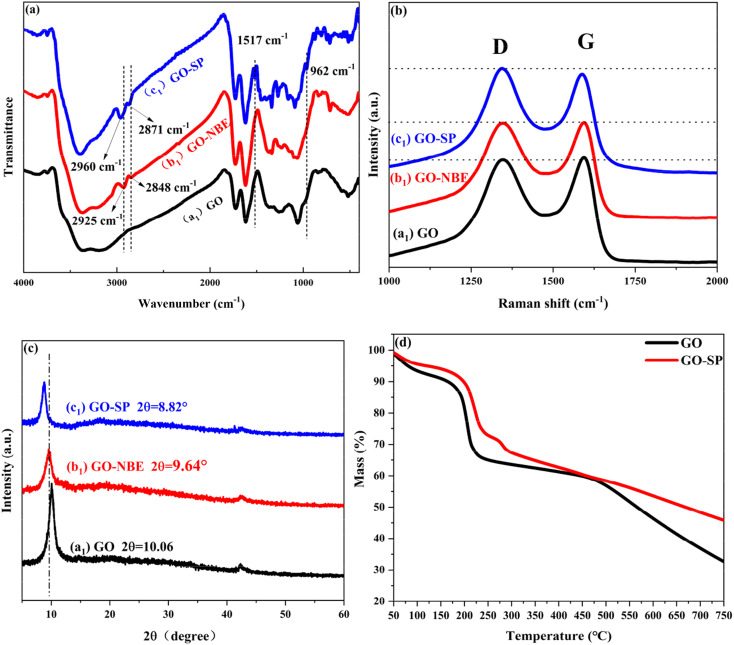
(a) FTIR spectra of (a_1_) GO, (b_1_) GO-NBE, and (c_1_) GO-SP, (b) XRD patterns of (a_1_) GO, (b_1_) GO-NBE and (c_1_) GO-SP, (c) Raman spectra of (a_1_) GO, (b_1_) GO-NBE and (c_1_) GO-SP, (d) TGA curves of GO and GO-SP.

FTIR spectroscopy as a practical effective measurement for qualitative testing was utilized to characterize the synthesis processes of the modified GO. The FTIR spectra of GO, GO-NBE, and GO-SP are shown in Fig. 2a. The characteristic absorption bands of GO are displayed at 3360 cm^−1^ for the –OH wide stretching vibration, 1726 cm^−1^ for the C=O stretching vibration, 1618 cm^−1^ for the C=C stretching vibration of the GO un-oxidized aromatic ring, 1050 cm^−1^ for the C–O–C stretching vibration, and 1228 cm^−1^ for the C–O–H stretching vibration. After the NBE modification, several new peaks of GO-NBE appeared at 2925 cm^−1^ and 2848 cm^−1^ attributed to the stretching vibration of C–H groups in NBE, which demonstrated the modification of NBE on the GO surface. The absorption peaks of GO-SP appeared as two obvious peaks at 1517 cm^−1^ (the stretching vibration of –NO_2_ on the benzene ring) and 962 cm^−1^ (the *trans*-C=C stretching vibration from the polymer chains of SPN) in the spectra. In addition, the peaks at 2960 cm^−1^ and 2870 cm^−1^ were derived from the –CH_3_ groups in spiropyran functionalized polymers. The results suggested that the SPN polymer (PSPN) bushes were grafted covalently onto the GO-NBE layers *via* the ROMP method.

Raman spectra further explained the change of the chemical structure in the process of GO modification as shown in Fig. 2b. The GO sample showed two obvious peaks of the D band at 1347 cm^−1^ and the G band at 1594 cm^−1^. The stronger D-band was caused by the symmetric stretching vibration of the sp^2^ carbon atoms in the aromatic ring, which requires defects. The G-band was arising due to the stretching vibrations between the sp^2^ hybridization of the carbon atoms. The G-band of GO-NBE and GO-SP showed a slight red shift in comparison with the GO spectra because of the increased defects in the covalent modification.^21^ The intensity ratio between D and G bands (*I*_D_/*I*_G_) is the parameter to describe the disorder of graphene. The *I*_D_/*I*_G_ values of GO, GO-NBE, and GO-SP were 0.979, 0.994, and 1.057 respectively. These values confirmed that more defects were created during the chemical grafting process. The structure of GO, GO-NBE, and GO-SP were further demonstrated by XRD analysis (Fig. 2c). The pure GO exhibited a sharp diffraction peak at 2*θ* = 10.06° with an interlayer distance of 0.879 nm according to Bragg's equation. The diffraction peak of GO-NBE shifted to 9.64° and the interlayer distance increased to 0.917 nm. The increased interlayer distance is caused by the modification of the NBE molecule between the GO layers. The diffraction peak of GO-SP shifted to 8.82°, and the interlayer distance increased to 1.002 nm. The SI-ROMP occurred in the GO layers and the grafted polymers grew between the layers of GO. The increased interlayer distance revealed the increase in the grafting ratio and this SI-ROMP modification method retained the ordered structure of the intercalated layers in GO. Fig. 2d displays TGA data for GO and GO-SP. GO experienced a significant weight loss in the temperature range from 150 to 210 °C, corresponding to the removal of oxygen-containing groups. In contrast, the TGA curves of the GO-SP material showed two distinct weightlessness at 220 °C and 280 °C, which correspond to the breakdown of the oxygen-containing groups on GO-SP and the degradation of polymers. Due to the degradation of the polymer carbon chain, GO-SP exhibited more significant weightlessness than GO at high temperatures (*T* > 490 °C) indicating that the surface of the GO sheet was grafted with polymers.

**Fig. 3:**
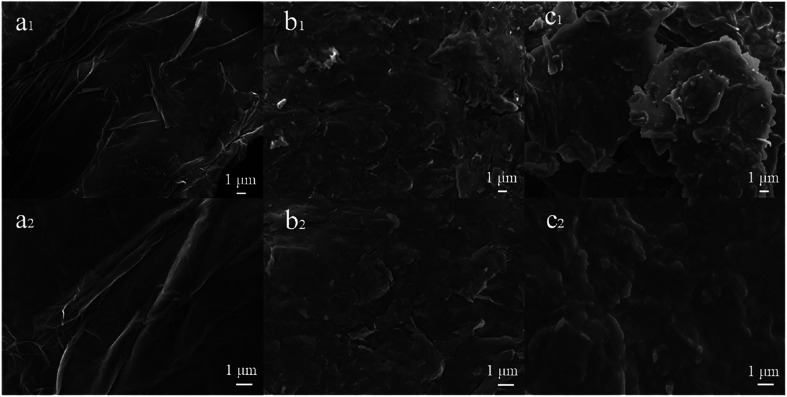
SEM images of GO (a_1_ and a_2_), GO-NBE (b_1_ and b_2_), and GO-SP (c_1_ and c_2_).

The surface morphologies of GO (a_1_ and a_2_), GO-NBE (b_1_ and b_2_), and GO-SP (c_1_ and c_2_) were observed using SEM (Fig. 3). The surface morphology of GO had an obvious wrinkled structure. Compared to GO, the surface of GO-NBE is rougher, which may be due to the covalent grafting of GO by small organic molecules (NBA). Furthermore, the GO-SP exhibited a rougher surface and the distribution of some polymer particles (C_2_) compared with GO-NBE on account of the PSPN aggregations on the GO sheets.

**Fig. 4:**
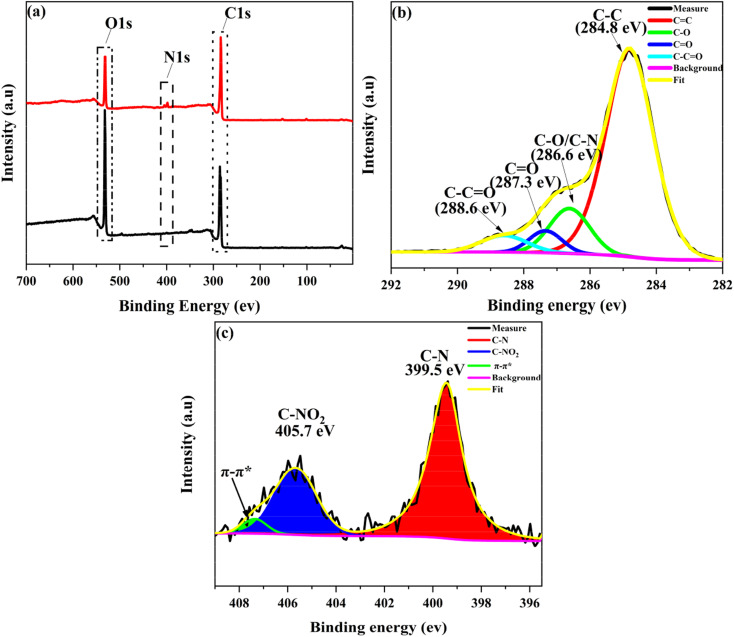
(a) XPS patterns of (a_1_) GO and (b_1_) GO-SP, (b) analysis of high-resolution C 1s XPS spectra of GO-SP, (c) analysis of high-resolution N 1s XPS spectra of GO-SP.

XPS survey spectra were further employed to characterize the composition and valence state of elements in GO-SP. As shown in Fig. 4, GO had two characteristic peaks of C 1s (286 eV) and O 1s (532 eV), and the C/O atomic ratio was 2.26. The C/O ratio of GO-SP increased to 3.99 after the modification of polymer brushes, which was attributed to the higher content of element C in the grafted polymer chains. The characteristic peak of the N 1s element in GO (0.97%) was less than that of the N 1s element in GO-SP (4.00%). The grafting ratio on the GO-SP was calculated using the equation:1
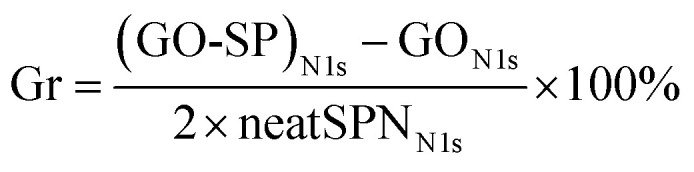
where (GO-SP)_N 1s_ is the nitrogen atomic percentage, GO_N 1s_ is the nitrogen atomic percentage, and neatSPN_N 1s_ is the nitrogen atomic percentage of SPN. Depending on the number of active sites on the anchored catalyst and the coverage density of the polymer, GO-SP received a better Gr value (64.19%). The increase of C and N elements indicated that graphene oxide is effectively and covalently modified by the PSPN polymer. The C 1s and N 2p core level spectra of GO-SP were peak-fitted to further clarify the chemical structure of the GO-SP surfaces and the result is shown in Fig. 4a. The C 1s core level spectrum of GO-SP was curve-fitted into four components with binding energies at 284.8, 286.6, 287.3 and 288.6 for C–C/C–H, C–O/C–N, C=O and O–C=O groups, respectively. In the N 1s core level spectrum of GO-SP (Fig. 4c), three peaks can be curve-fitted for N–C, NO_2_–C, and π–π* at binding energies of 399.5 eV, 405.7 eV, and 407.4 eV, respectively, owing to the structure of PSPN. The increase of the C/O ratio of GO-SP and the fitting peak of C 1s and N 2p core level spectra offered further elucidation on the functionalization of graphene oxide by the SPN polymer brushes.

### 3.2 Photochromic properties of GO-SP

**Fig. 5:**
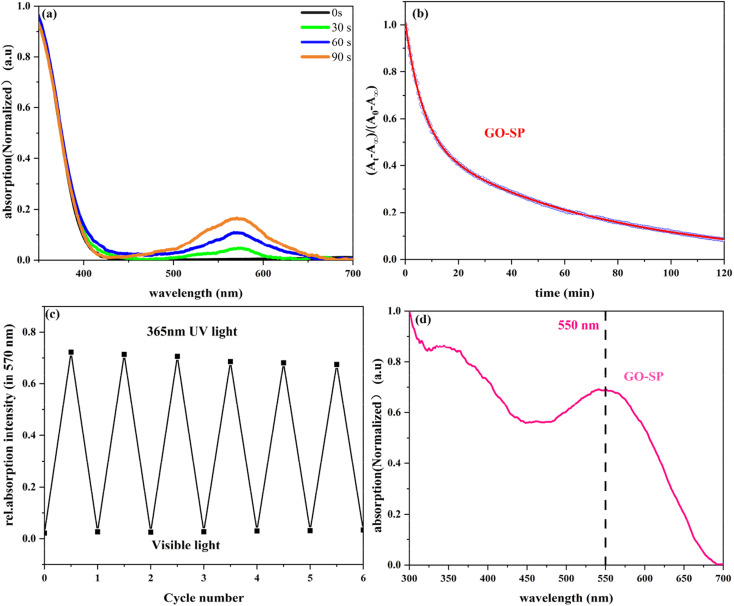
Photochromic properties of GO-SP: (a) UV-vis absorption spectra (smoothened and normalized to exclude the effect of the UV-irradiation lamp) of GO-SP with different UV irradiation times (30 s, 90 s, and 50 s), (b) bi-exponential fit of the bleaching kinetics about GO-SP in the dark, (c) reversibly photoresponse of the GO-SP and (d) UV-vis diffuse reflection (UV-vis DRS) of GO-SP.

GO-SP was dispersed in dimethylformamide (DMF) and treated at different irradiation times under UV light, as shown in Fig. 5a. The spiro bond of C–O in the SP form grafted on the GO surface underwent bond cleavage after UV irradiation, and transformed into the MC form. The MC form (GO-MC) showed a strong absorption band at about 570 nm, and this absorption band was enhanced with the increase of the irradiation time. When GO-MC was irradiated by visible light (>500 nm), the structure reverted to the ring-closed form (GO-SP) with more thermodynamic stability.^35^ Meanwhile, GO-SP nanoparticles showed a strong absorption at 550 nm under the condition of ultraviolet irradiation (Fig. 5d). The kinetic attenuation curve of MC form was measured from the absorption at 570 nm under the dark conditions (Fig. 5b), which fitted the bi-exponential decay model (eqn (2)).^36^ The half-life of GO-MC was approximately 38 min, and the fading process was not completed after 2 h in the dark condition. Alternating UV irradiation (90 s) and visible light (360 s) was employed to investigate the photochromic behavior and fatigue resistance of GO-SP and the absorption at 570 nm was measured as shown in Fig. 5c. The photochromic behavior of GO-SP maintained higher stability, but the absorption of the GO-MC decreased after each cycle. There was only a 6.5% loss of absorption intensity within 6 conversion cycles. The results showed that the film had good optical reversibility.^37^

2 (*A*_*t*_ − *A*_∞_)/(*A*_0_ − *A*_∞_) = *F* exp(−*k*_1_*t*) + (1 − *F*) exp(−*k*_2_*t*)

**Fig. 6:**
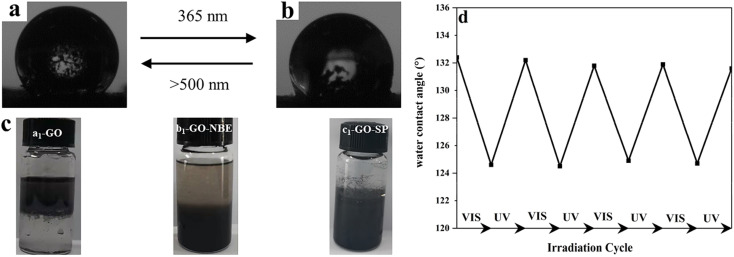
(a) Water contact angle of GO-SP after visible light illumination, (b) the water contact angle of GO-SP after UV irradiation, (c) dispersity of (a_1_) GO, (b_1_) GO-NBE, and (c_1_) GO-SP in CH_2_Cl_2_/H_2_O after standing of the ultrasonic dispersion for 3 days, (d) the water contact angle of GO-SP upon sequential irradiation with UV (365 nm) light and visible light.

The light-induced contact angles of GO-SP were employed to characterize the reversible changes of surface energy upon irradiation of light. The spiro bond of C–O in the SP form underwent bond cleavage and transformed into the ring-opened MC form. As shown in Fig. 6b, the increase of the surface energy led to the decrease of water contact angle (124°) after UV irradiation. The water contact angle returned to 132° (Fig. 6a) after visible light illumination. The contact angle dropped by 8° in this process and the switching was reversible for at least 5 cycles (Fig. 5c). The two states of GO-SP nanoparticles had better stability in the cycling possess between the UV and visible light. The effectiveness of covalent modification can be confirmed by the dispersity transformation of GO in an organic solvent (Fig. 6d). Both NBE and SPN are hydrophobic and can be dissolved in common organic solvents. GO, GO-NBE, and GO-SP were dispersed homogeneously in a mixture of CH_2_Cl_2_ and H_2_O. GO was dispersed in the upper layer (H_2_O) of the mixed solvent for a long time. On the contrary, GO-NBE and GO-SP exhibited better hydrophobicity and could be dispersed in the lower layer (CH_2_Cl_2_) of the mixed solvent. The modification of NBE and SPN improved the solubility of GO sheets from hydrophilic to hydrophobic.^21^

## 4. Conclusions

Based on the method of SI-ROMP using norbornene derivatives as monomers, spiropyran-functionalized polynorbornene was grafted onto the surface of GO to prepare photochromic spiropyran polymer brushes. The grafting rate of the functionalized polymer brushes was calculated as 64.19% from the XPS survey spectra. GO-SP showed good photochromic behavior demonstrated by UV-vis absorption results. The GO-SP solid exhibited rapid coloring (in 90 s)/bleaching (in 360 s) phenomenon and maintained relatively stable photochromic performance in consecutive UV/dark cycles. Besides the photochromic GO having significant scientific relevance for nanomaterial materials, it could also find application prospects in photoresponse, coating, and switches.

## Author contributions

BangSen Li: conceptualization, investigation, writing original draft. Wenya Zhu: conceptualization, formal analysis, methodology. Jinrui Liu: investigation, validation. Shishu Sun: formal analysis, supervision. Yan Zhang: conceptualization, validation, investigation. DaShuai Zhang: supervision. Chen Li: formal analysis, supervision. Jianjun Shi: conceptualization, project administration, writing review & editing. Zaifeng Shi: conceptualization, project administration, writing review & editing.

## Conflicts of interest

The authors declare that they have no known competing financial interests or personal relationships that could have appeared to influence the work reported in this paper.

## Supplementary Material
